# Supplementary material for: Cryo-EM structure of a licensed DNA replication origin
Source: Nat Commun. 2017 Dec 21;8:2241. doi: 10.1038/s41467-017-02389-0 (PMC5740162; doi:10.1038/s41467-017-02389-0)
Supplement: Supplementary file 1 — Supplementary Information [file 41467_2017_2389_MOESM1_ESM.pdf]

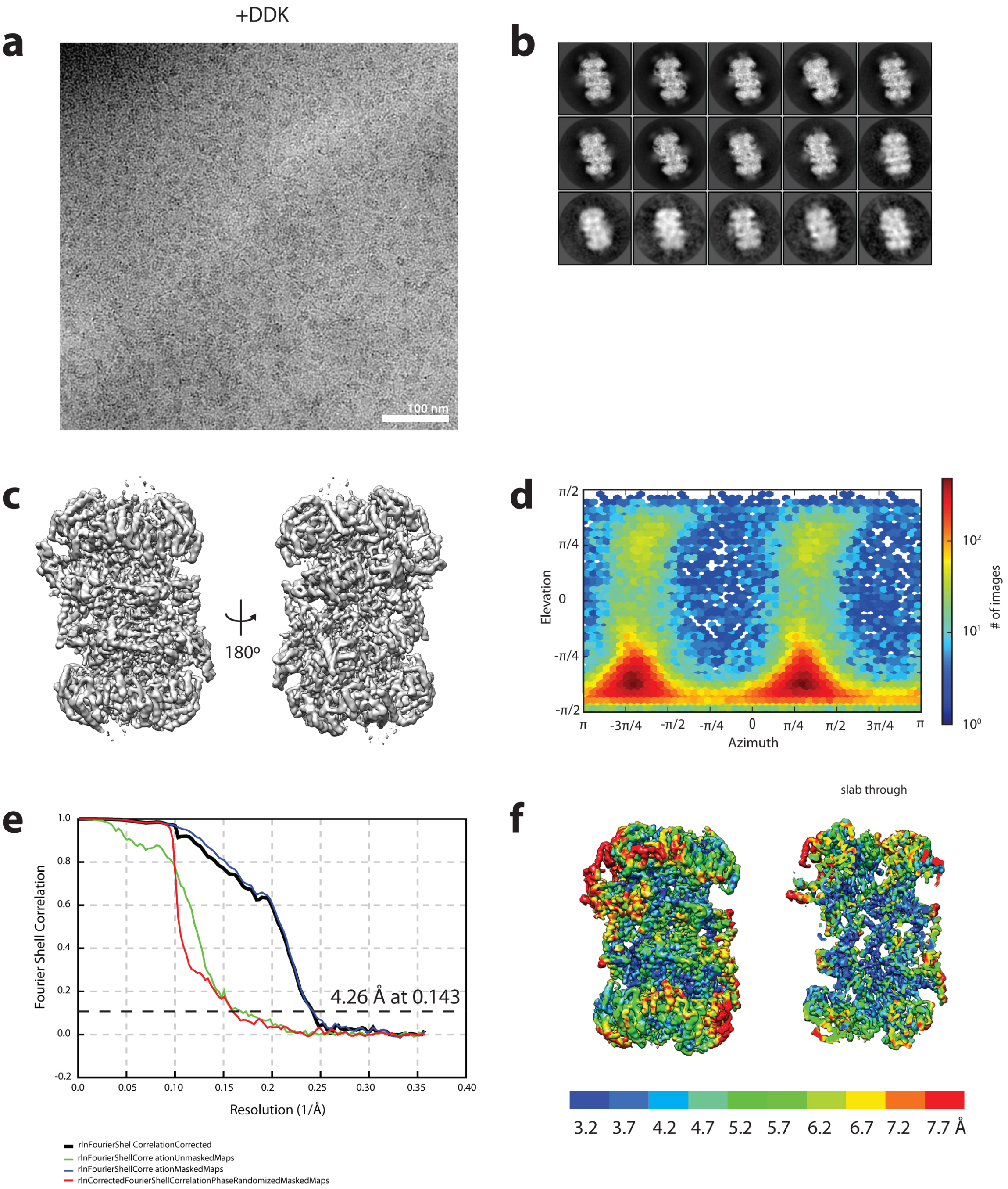

Supplementary Figure 1: Cryo-electron microscopy of the DDK phosphorylated MCM double hexamer. (a) Cryo-electron micrograph acquired with a K2 summit camera operated in counting mode. (b) 2D class averages after one round of cleaning. (c) 3D volume shown in two views. (d) Angular distribution. (e) Gold standard fourier shell correlatation using the 0.143 criterion. (f) Local resolution as determined by ResMap[1].

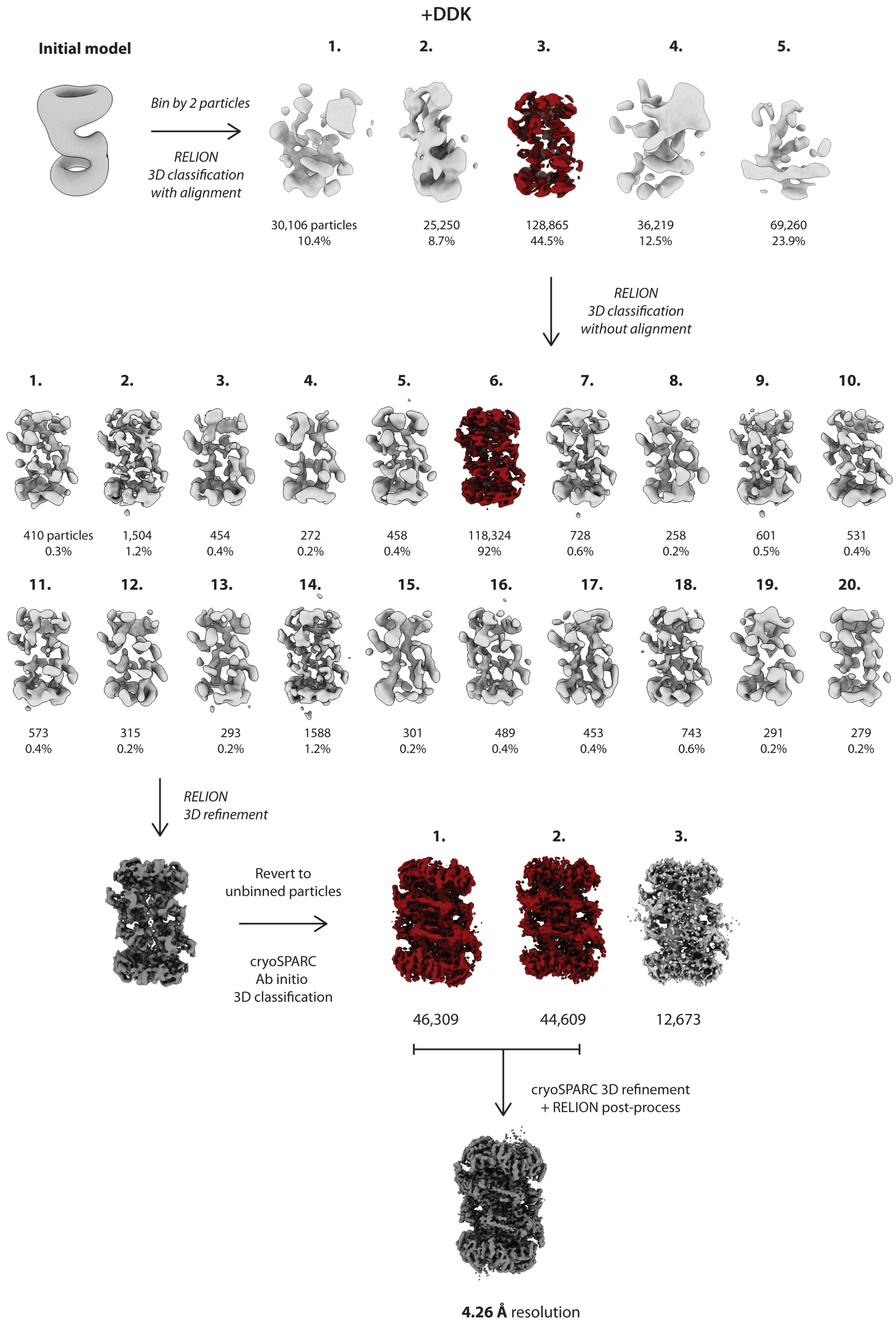

Supplementary Figure 2: Overview of image processing for the DDK phosphorylated MCM double hexamer. Particles from 3D classes indicated in red were selected for further ab-initio 3D classification and refinement in cryoSPARC[2].

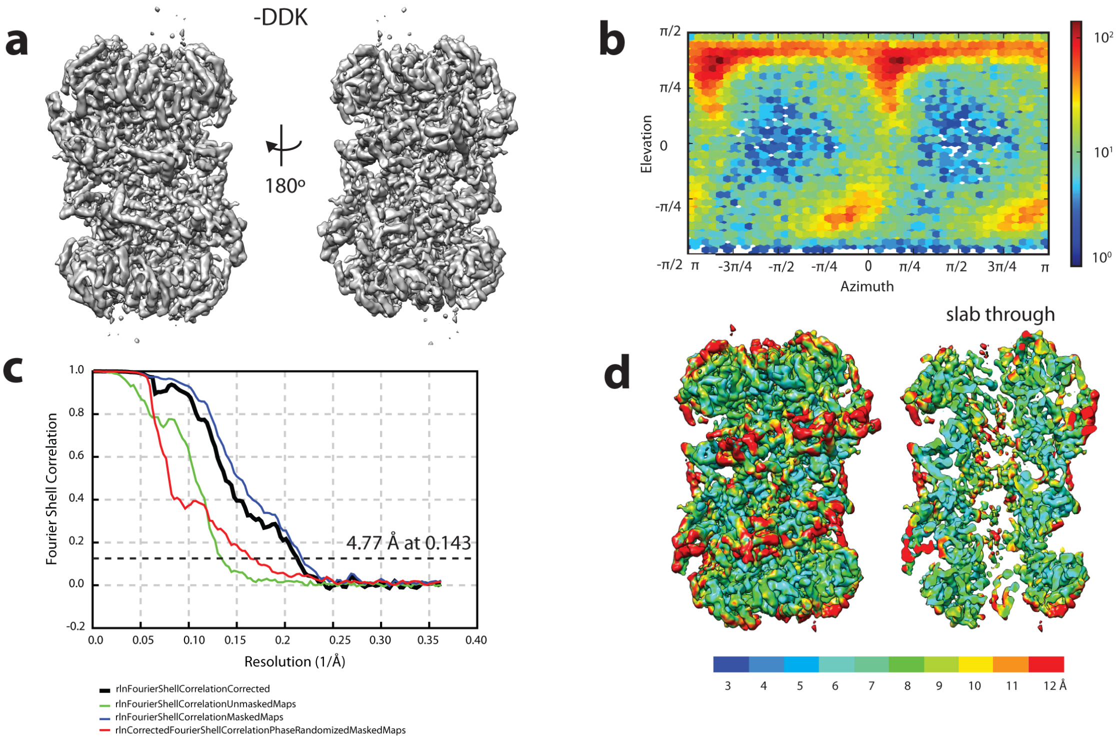

Supplementary Figure 3: Cryo-electron microscopy of the unmodified MCM double hexamer. (a) 3D volume shown in two views. (b) Angular distribution. (c) Gold standard fourier shell correlation using the 0.143 criterion. (d) Local resolution as determined by ResMap[1].

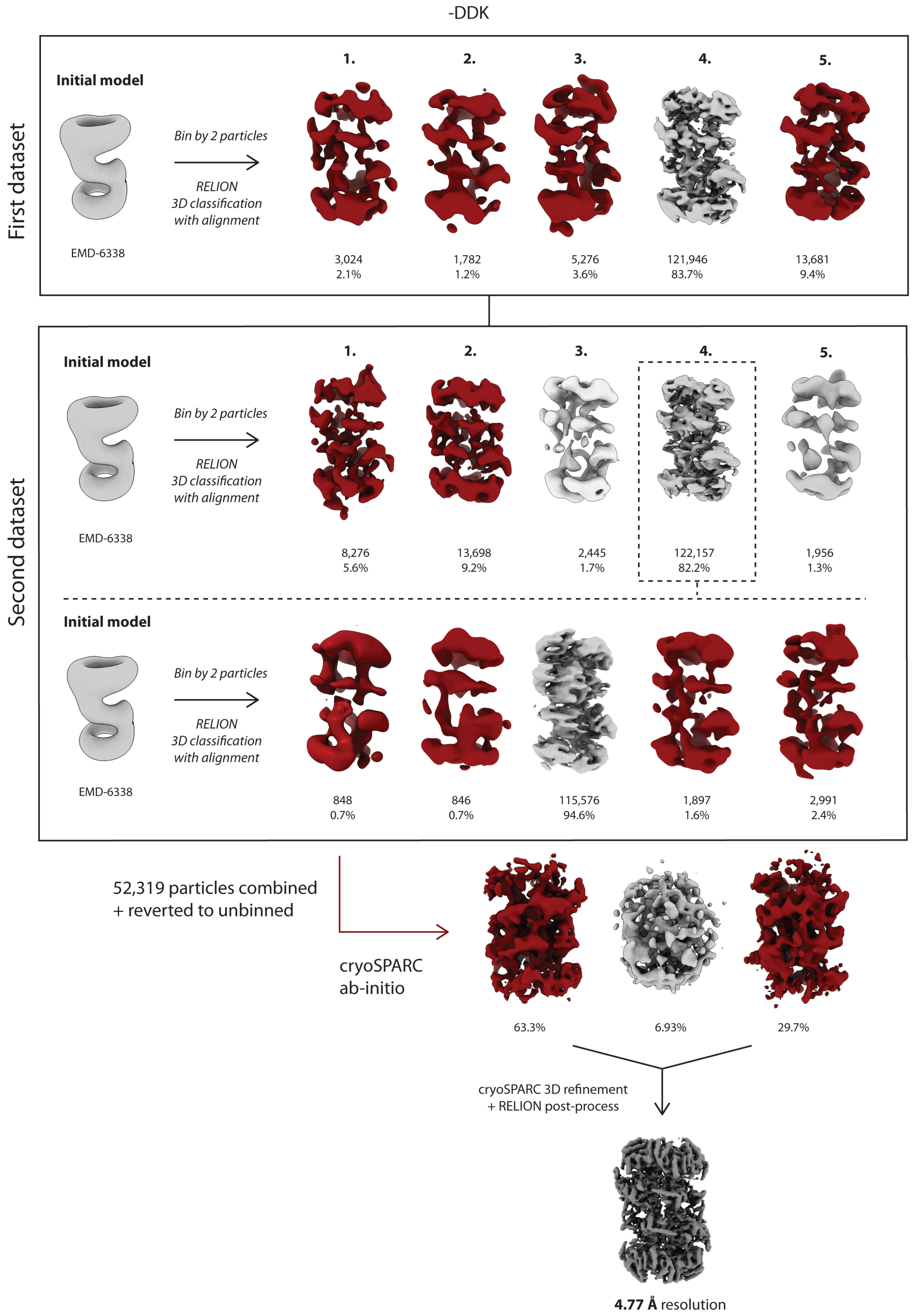

Supplementary Figure 4: Overview of image processing for the unmodified MCM double hexamer. Particles from isotropic 3D classes were selected (coloured red) and combined from two different datasets for ab-initio classification and refinement in cryoSPARC[2].

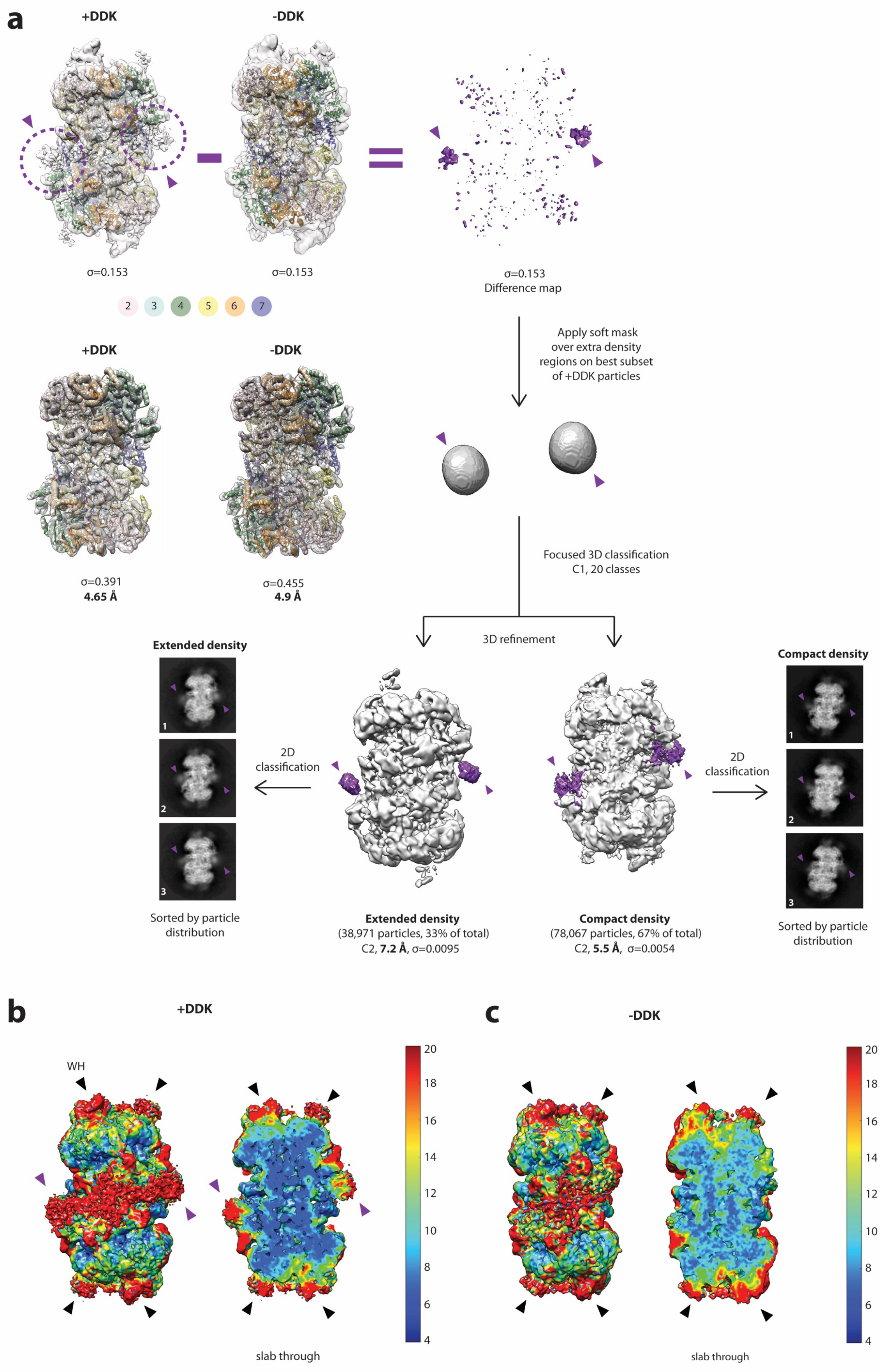
a
+DDK
-DDK
σ=0.153
σ=0.153
σ=0.153
Difference map
2 3 4 5 6 7
Apply soft mask
over extra density
regions on best subset
of +DDK particles
+DDK
-DDK
σ=0.391
4.65 Å
σ=0.455
4.9 Å
Focused 3D classification
C1, 20 classes
3D refinement
Extended density
2D
classification
Sorted by particle
distribution
Extended density
(38,971 particles, 33% of total)
C2, 7.2 Å, σ=0.0095
Compact density
(78,067 particles, 67% of total)
C2, 5.5 Å, σ=0.0054
Compact density
2D
classification
Sorted by particle
distribution
b
+DDK
WH
slab through
20 18 16 14 12 10 8 6 4
c
-DDK
slab through
20 18 16 14 12 10 8 6 4

Supplementary Figure 5: DDK phosphorylation partially stabilises the MCM N-terminal tails of Mcm4/6. (a) Unsharpened, DDK phosphorylated and unmodified MCM maps, with docked atomic coordinates, displayed at low and high contour levels (first and second row, respectively). Purple arrows and dashed circle point to additional density observed in the phosphorylated double hexamer at low contour level. This density is absent in the unmodified double hexamer at matching contour level. A difference map (purple) was generated by subtracting the unmodified from the DDK-phosphorylated double-hexamer map. The difference map was low-pass filtered and used to generate a soft mask for further local analysis. Following asymmetric focused 3D classification of the DDK phosphorylated double hexamer particles, two major conformations of extra density were identified, which we describe as “extended” and “compact”. Because these densities appeared symmetrical, particles contributing to the two conformers were separately used for refining a 3D structure in C2, to a resolution of 7.2 Å (extended state) and 5.5 Å (compact state). 2D classification of the two subsets of particles (non-symmetrised) confirms the presence of the two conformations (also see Supplementary Movie 2). All image processing steps were performed in Relion 2.0[3]. (b) Local resolution of DDK-phosphorylated and unmodified MCM double hexamer, as determined by ResMap[1]. Phospho-Mcm4/6 Nt have similar resolution as the flexible winged-helix domains decorating the ATPase tier. These flexible C-terminal elements are known to become visible in the activated CMG[4]. Likewise, the flexible phospho Mcm4/6 Nt elements might become more rigid during subsequent stages of replisome maturation (e.g. Sld3/7/Cdc45 recruitment).

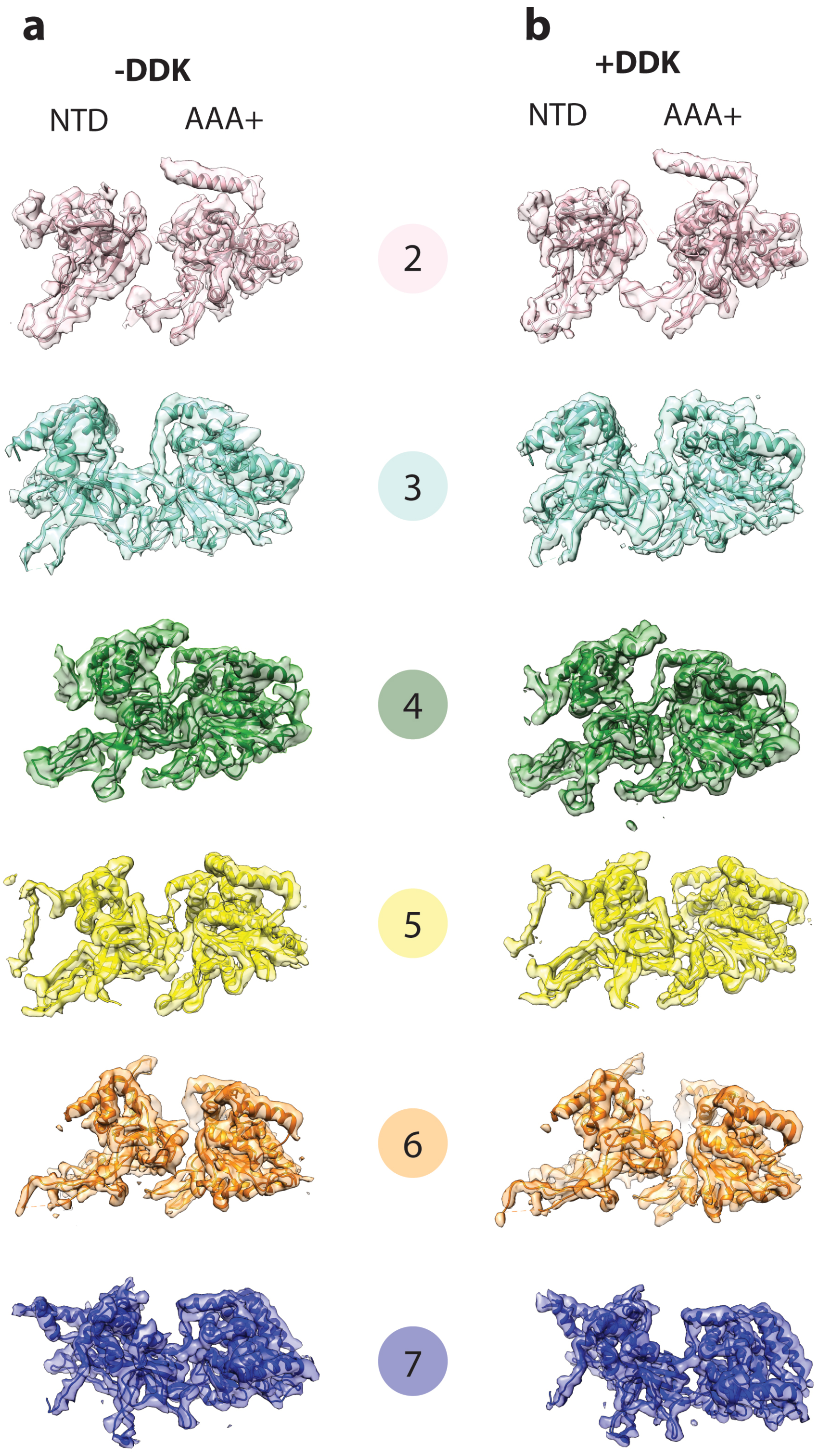

Supplementary Figure 6: Segmented MCM density of the (a) unmodified (-DDK) and (b) DDK phosphorylated double hexamers with docked coordinates (PDB: 3ja8). No major conformational change is observed in MCM double hexamer upon DDK phosphorylation (see also Supplementary Movie 1).

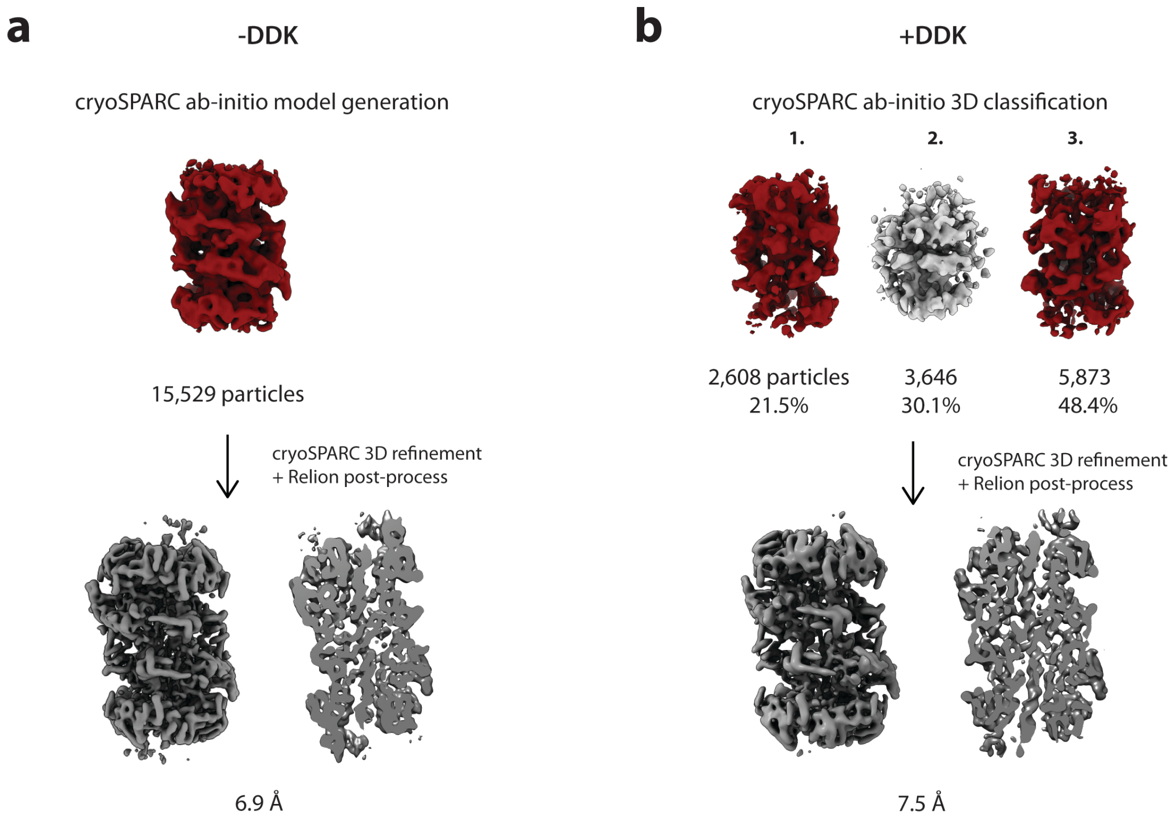

Supplementary Figure 7: 3D ab-initio model generation and 3D refinement of DNA bound double hexamer particles with the (a) unmodified and (b) DDK phosphorylated forms in cryoSPARC[2].

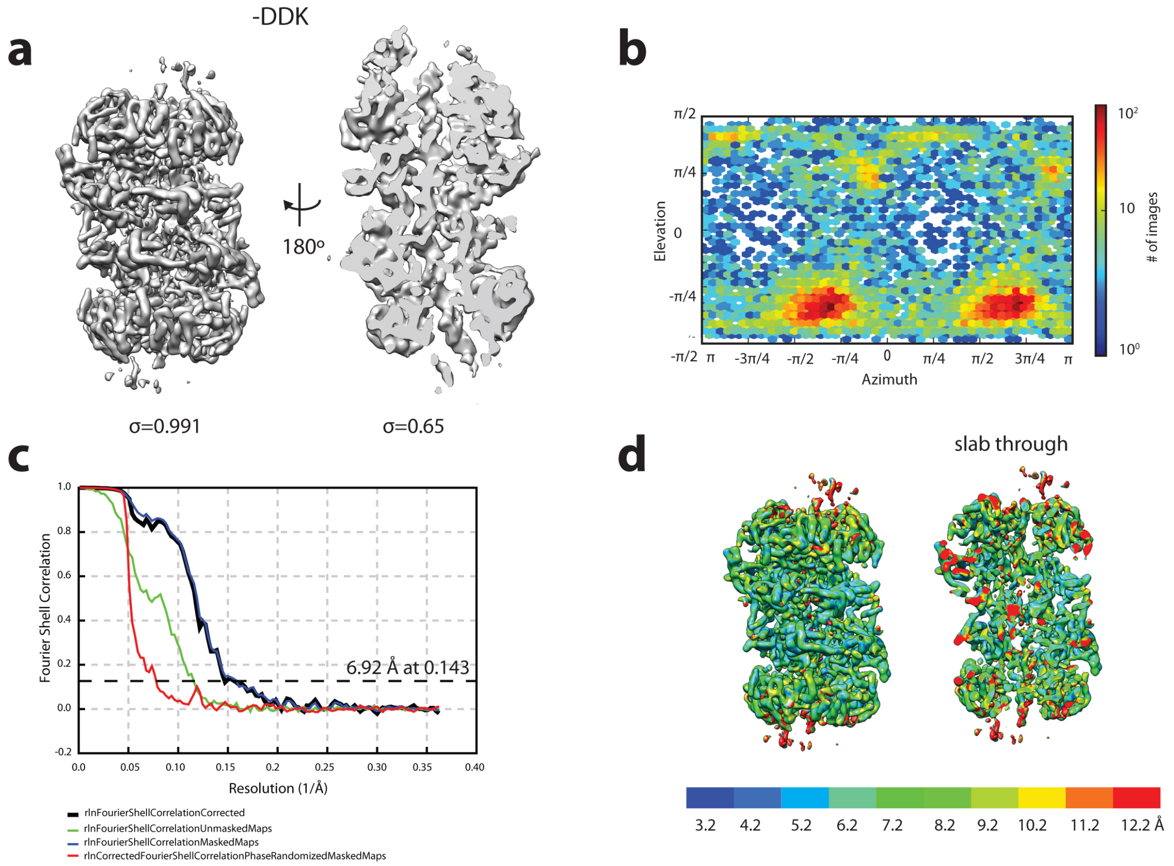

Supplementary Figure 8: Map, angular distribution and resolution estimation for the DNA bound unmodified MCM double hexamer. (a) 3D reconstruction in full surface and slab view at lower contour level. (b) Angular distribution. (c) Gold standard fourier shell correlation using the 0.143 criterion. (d) Local resolution as determined by ResMap[1].

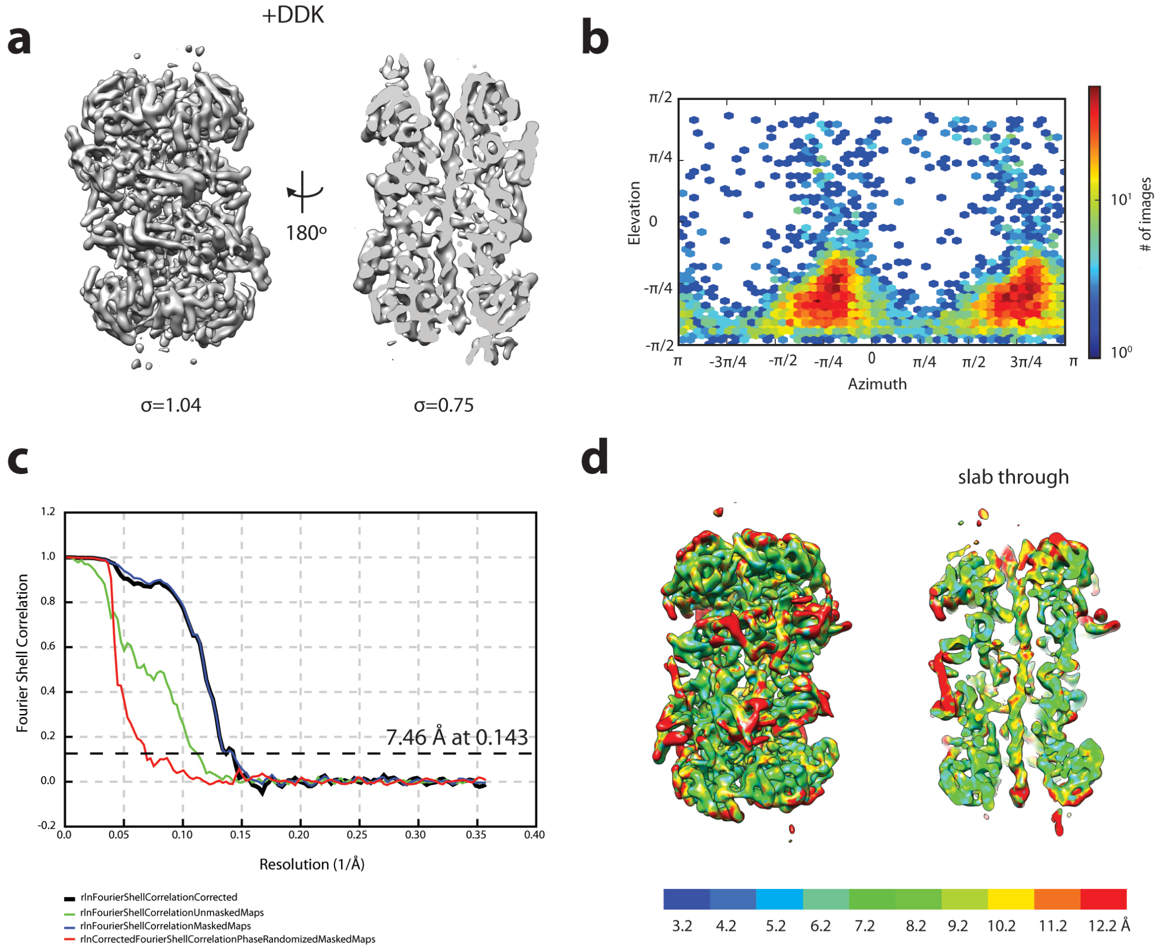

Supplementary Figure 9: Map, angular distribution and resolution estimation for the DNA bound DDK phosphorylated MCM double hexamer. (a) 3D reconstruction in full surface and slab view at lower contour level. (b) Angular distribution. (c) Gold standard fourier shell correlation using the 0.143 criterion. (d) Local resolution as determined by ResMap[1].

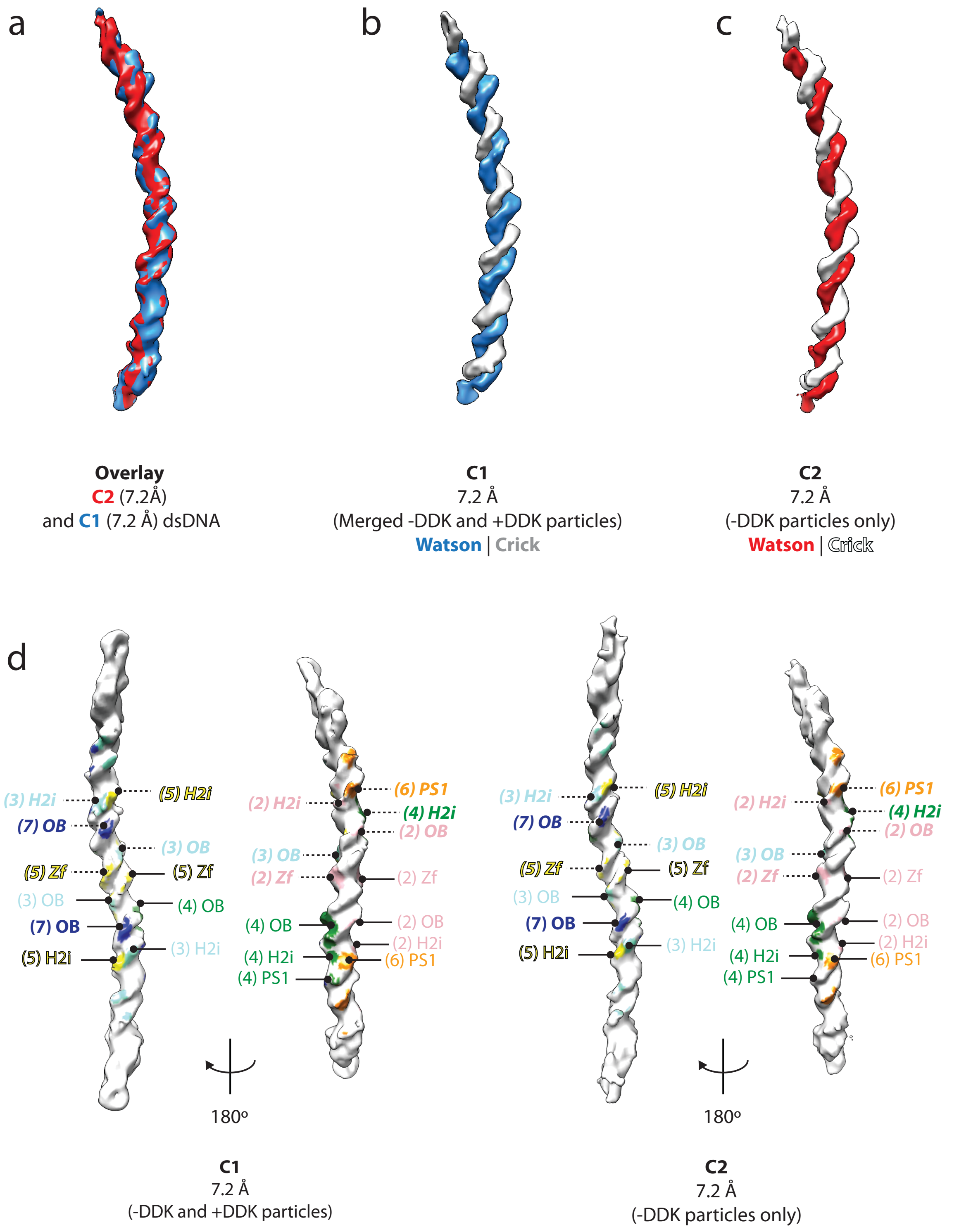

Supplementary Figure 10: DNA arrangement and protein-DNA contacts in the double hexamer are preserved regardless of symmetry imposition. To match the same resolution as the C2 unmodified double hexamer map (7.2 Å), DDK phosphorylated and unmodified datasets were merged for the C1 reconstruction. This is possible because no conformational change is detected in the MCM core upon phosphorylation. (a) Superposition of extracted DNA density from asymmetric (blue) and symmetric reconstructions (red), both at 7.2 Å. (b) DNA density from asymmetric reconstruction with segmented Watson (blue) and Crick (grey) strands. (c) DNA density from C2 reconstruction with segmented Watson (red) and Crick (white) strands. (d) MCM-DNA contacts are displayed as a patch of colour on extracted

DNA density from asymmetric and symmetric reconstructions. Color zone function in UCSF Chimera[5] was used to colour the DNA density, according to the selected MCM polypeptide coordinates (not shown for clarity). A colouring radius of 4 was applied on both C1 and C2 DNA densities. H2i, Helix-2 insert. OB, oligonucleotide/oligosaccharide-binding fold. PS1, pre-sensor 1 hairpin. Zf, Zinc Finger.

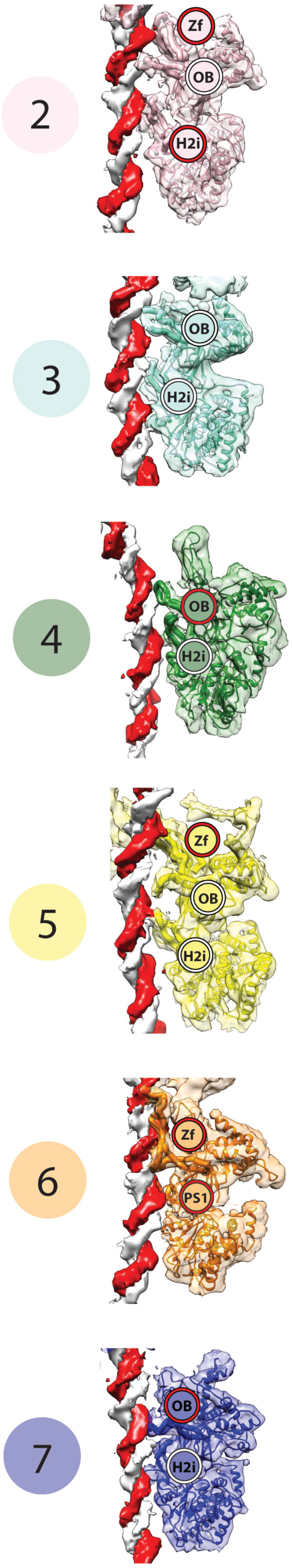

Supplementary Figure 11: Protein-DNA contacts in the MCM double hexamer. OB, oligosaccharide/oligonucleotide fold. PS1, Pre-sensor 1 hairpin. Zf, zinc-finger domain. H2i, helix-2-insert.

Supplementary Table 1: Data collection and image processing parameters.

	**+DDK**	**+DDK**	**-DDK**
**Institution**	NeCEN	Diamond (eBIC)	Crick (Cryo-EM STP)
**Detector**	K2 summit	K2 summit	K2 summit
**Frames**	29	28	30
**Exposure (s)**	5.8	14	12
**Dose (e^-/Å^2)**	50	50	50
**Micrographs**	3,996	1,974	8,331
**Pixel size (Å)**	1.4	1.36	1.38
**Raw particles**	299,604	183,221	582,423
**Final particles ensemble structure**	90,918	78,821	52,319
**Final particles DNA-bound structure**	8,343	3,784	15,529
**Resolution ensemble structure**	4.26 Å	5.33 Å unsharpened, map anisotropic (not shown)	4.77 Å
**Resolution DNA-bound**	7.46 Å		6.92 Å

Supplementary References

1. Kucukelbir, A., Sigworth, F.J. & Tagare, H.D. Quantifying the local resolution of cryo-EM density maps. *Nat Methods* **11**, 63-5 (2014).
2. Punjani, A., Rubinstein, J.L., Fleet, D.J. & Brubaker, M.A. cryoSPARC: algorithms for rapid unsupervised cryo-EM structure determination. *Nat Methods* **14**, 290-296 (2017).
3. Kimanius, D., Forsberg, B.O., Scheres, S.H. & Lindahl, E. Accelerated cryo-EM structure determination with parallelisation using GPUs in RELION-2. *Elife* **5**(2016).
4. Yuan, Z. et al. Structure of the eukaryotic replicative CMG helicase suggests a pumpjack motion for translocation. *Nat Struct Mol Biol* **23**, 217-24 (2016).
5. Pettersen, E.F. et al. UCSF Chimera--a visualization system for exploratory research and analysis. *J Comput Chem* **25**, 1605-12 (2004).
