# Supplementary material for: Cryo-EM structure of a licensed DNA replication origin
Source: Nat Commun. 2017 Dec 21;8:2241. doi: 10.1038/s41467-017-02389-0 (PMC5740162; doi:10.1038/s41467-017-02389-0)
Supplement: Supplementary file 3 — Description of Additional Supplementary Files [file 41467_2017_2389_MOESM3_ESM.pdf]

Description of Additional Supplementary Files

File Name: Supplementary Movie 1

Description: Comparison between the unmodified and DDK-treated MCM. A molecular morph between the non-phosphorylated and phosphorylated MCM shows negligible conformational transitions in the MCM core particle and no change in nucleotide occupancy in the ATPase active sites.

File Name: Supplementary Movie 2

Description: 2D classification of DDK phosphorylated MCM double hexamers reveal a conformational change of the phospho-Mcm4/6 tails.

File Name: Supplementary Movie 3

Description: A mechanism for duplex-DNA untwisting upon MCM-to-CMG transition. MCM morphing to CMG alters duplex-DNA interactions with the N-terminal MCM domain. This transition is accompanied by substrate rotation and duplex-DNA underwinding. Inspection of the MCM side view highlights stretching of the ATPase captured strand, occurring between the N-terminal and AAA+ pore loops.
